# Relation between Morphology and Porous Structure of SAPO-11 Molecular Sieves and Chemical and Phase Composition of Silicoaluminophosphate Gels

**DOI:** 10.3390/gels8030142

**Published:** 2022-02-24

**Authors:** Marat R. Agliullin, Roman E. Yakovenko, Yury G. Kolyagin, Dmitry V. Serebrennikov, Farkhad S. Vildanov, Tatyana R. Prosochkina, Boris I. Kutepov

**Affiliations:** 1 Institute of Petrochemistry and Catalysis, Ufa Federal Research Centre of the Russian Academy of Sciences (UFRC RAS), 450075 Ufa, Russia; kutepoff@inbox.ru; 2 Faculty of Chemical Engineering, Ufa State Petroleum Technological University, 450062 Ufa, Russia; stratpro@yandex.ru (F.S.V.); agidel@ufanet.ru (T.R.P.); 3 Research Institute “Nanotechnologies and New Materials”, M.I. Platov South-Russian State Polytechnic University, 346428 Novocherkassk, Russia; jakovenko39@gmail.com; 4 Department of Chemistry, Moscow State University, 119991 Moscow, Russia; kolyagin@mail.ru

**Keywords:** silicoaluminophosphate gels, zeolites, SAPO-11, crystal morphology, micro-mesoporous materials

## Abstract

The formation of silicoaluminophosphate gels using boehmite, Al isopropoxide, and di-n-propylamine as a template of silicoaluminophosphate gels as well as their subsequent crystallization into SAPO-11 molecular sieves was studied in detail using X-ray fluorescence spectroscopy (XRF), powder X-ray diffraction (XRD), Raman spectroscopy, scanning electron microscopy (SEM), transmission electron microscopy (TEM), and N_2_ adsorption–desorption methods. The effect of the chemical and phase composition of silicoaluminophosphate gels on the physicochemical properties of SAPO-11 molecular sieves was shown. The secondary structural units that the AEL lattice is composed of were found to be formed at the initial stage of preparation involving aluminum isopropoxide. Several approaches to control their morphology and secondary porous structure are also proposed.

## 1. Introduction

The current progress in oil refining and petrochemical industries is largely associated with the use of zeolites in the development of modern catalysts and adsorbents [1,2]. The widespread application of zeolites in the chemical industry is based on a successful combination of strong Brønsted acid sites and a developed microporous structure providing a molecular sieve effect [3]. As a rule, most of the zeolites employed in industrial processes are aluminosilicates in terms of chemical composition.

In 1984, Wilson et al. first reported on the synthesis of SAPO-n silicoaluminophosphate molecular sieves [4,5,6]. SAPO-n are characterized by a wide variety of structures differing in pore size (SAPO-18 3.8 × 3.8 Å, SAPO-5 7.3 × 7.3 Å, VPI-5 12.7 × 12.7 Å) and channel dimensions (1D SAPO-11, 2D SAPO-40, 3D SAPO-50) [7]. The Brønsted and Lewis acid sites in SAPO-n are formed as a result of the isomorphic insertion of silicon atoms into the aluminophosphate lattice during crystallization.

Currently, molecular sieves based on SAPO-n silicoaluminophosphates are already used in industry. In particular, UOP and the Dalian Institute of Chemical Physics (DICP) have developed and implemented industrial processes on the basis of SAPO-34 to obtain lower olefins from methanol [8]. Chevron has introduced a process for isodewaxing oils on the basis of SAPO-11 [9,10].

Among the wide variety of SAPO-n, SAPO-11 molecular sieves (AEL structure) are of particular interest due to the presence of a one-dimensional channel system with elliptical pores of 4.0 × 6.5 Å, which are comparable to the molecular sizes of various practically important compounds and moderately strong acid sites. SAPO-11 molecular sieves are the most selective catalysts for hydroisomerization of C_7+_ n-paraffins [11,12,13], isomerization of n-butene to isobutylene [14] and cyclohexanone oxime to caprolactam [15,16] as well as the methylation of aromatic hydrocarbons [17,18].

Despite some success achieved in the synthesis and application of SAPO-11 molecular sieves in catalysis, the issues of formation and the relation between the results of subsequent crystallization of reaction gels and their physicochemical properties have been poorly studied thus far. Addressing these issues will make it possible to specify SAPO-11 crystallization and proceed to the development of these materials with the desired properties, which is one of the current challenges in the field of synthesis and research of molecular sieves.

Already in [19], phosphoric acid was found to react predominantly with an amine in the preparation of aluminophosphate reaction gels using boehmite as a source of Al. In this case, a gel of a complex composition is formed. However, the chemical and phase composition and their influence on the subsequent crystallization of AlPO_4_-11 were not studied in this work.

It was shown in [20] that, upon stirring the gel at room temperature for up to 3 h, non-porous tridymite is formed in the process of further crystallization. Increasing the duration to 6 h results in the formation of SAPO-11 of high phase purity in a higher yield.

Earlier [16,21,22], we demonstrated that the reactivity of the Al source had a significant impact on the crystal morphology and secondary porous structure of the forming AlPO_4_-11 molecular sieves.

The research results prove the importance of the gel formation stage during subsequent crystallization of the above materials. Therefore, we carried out a detailed study of the relation between the chemical and phase composition of silicoaluminophosphate gels prepared using various sources of aluminum and different contents of the template, and the morphology and porous structure of SAPO-11 molecular sieves formed during crystallization.

## 2. Results and Discussion

Table 1 and Table 2 detail the chemical composition of the reaction gels and crystallization products based on them. The silicon content in the initial gels turned out to be higher than in the crystallization products. The fact that some of the silicon is not incorporated into the crystal lattice and remains in the mother liquor after crystallization accounts for the results obtained.

**Figure 1:**
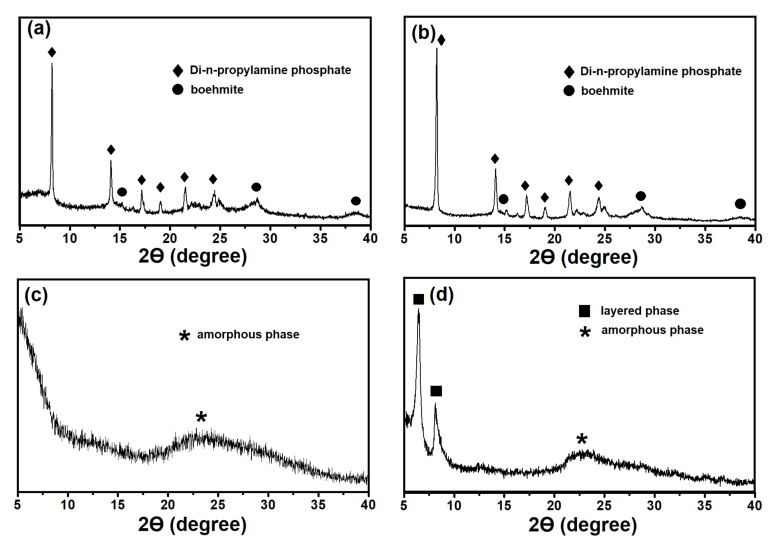
X-ray diffraction patterns of the silicoaluminophosphate gels: (**a**) Sample SAPO-PB-1.0; (**b**) Sample-SAPO-PB-1.5; (**c**) Sample-SAPO-iAl-1.0; (**d**) Sample-SAPO-iAl-1.5.

Figure 1 illustrates the X-ray diffraction patterns of dried gels; the phase composition is given in Table 1. In fact, the samples of gels prepared using boehmite, regardless of the content of the template, are a mixture of phases of di-n-propylamine phosphate and undissolved boehmite. The results indicate a weak interaction between sources of aluminum and phosphorus at the initial stage of the preparation of reaction gels. The gel samples prepared using Al isopropoxide, depending on the template content, can either be amorphous (SAPO-iAl-1.0 sample) or a mixture of phases of crystalline layered and amorphous silicoaluminophosphate (SAPO-iAl-1.5 sample). 

It should be noted that the pH value of the gels strongly depends on the reactivity of the Al source and the content of the template. Thus, when preparing gels using the more reactive Al isopropoxide, when aluminophosphate is formed already at the initial stages of their formation, the pH value exceeds ~7. When using a less reactive boehmite, it is ~5, even at a DPA/Al_2_O_3_ ratio of 1.5.

**Figure 2:**
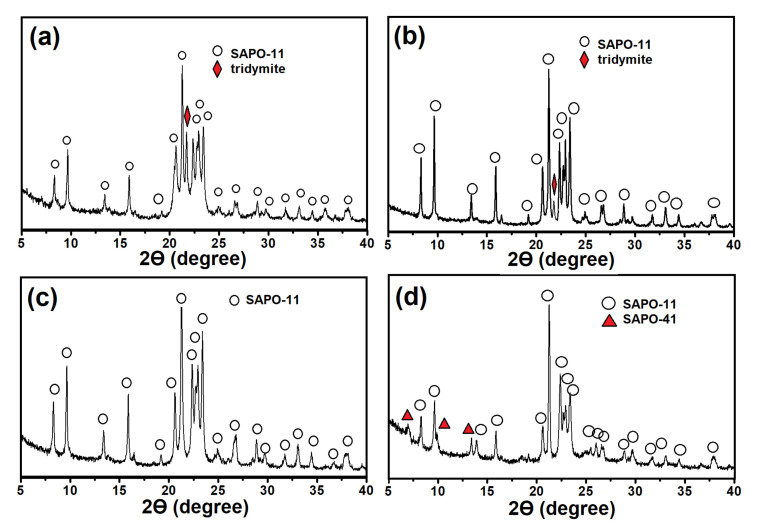
X-ray diffraction patterns of the products of crystallization of silicoaluminophosphate gels: (**a**) Sample SAPO-11-PB-1.0; (**b**) Sample-SAPO-11-PB-1.5; (**c**) Sample-SAPO-11-iAl-1.0; (**d**) Sample-SAPO-11-iAl-1.5.

Differences in the phase composition of gels affect the phase composition of the products of their crystallization (Figure 2). The SAPO-11 phase with an admixture of non-porous tridymite is formed from the samples of gels prepared on the basis of boehmite, regardless of the template content. During crystallization of the gels prepared using aluminum isopropoxide, only molecular sieves are formed. Meanwhile, SAPO-11 of high phase purity is formed from the SAPO-iAl-1.0 gel, while SAPO-11 with SAPO-41 impurities is formed from the SAPO-iAl-1.5 gel.

Thus, crystallization of SAPO-11 of high phase purity is possible only from amorphous SAPO-11-iAl-1.0 gel under DPA/Al_2_O_3_ ratio = 1.0. When it increases to 1.5, the conditions favorable for the formation of SAPO-41 are achieved. It is important to note that in the amorphous and layered phase, the sources of aluminum and phosphorus almost completely reacted with each other with the formation of bonds of the Al–O–P type, in contrast to gels prepared using boehmite.

**Figure 3:**
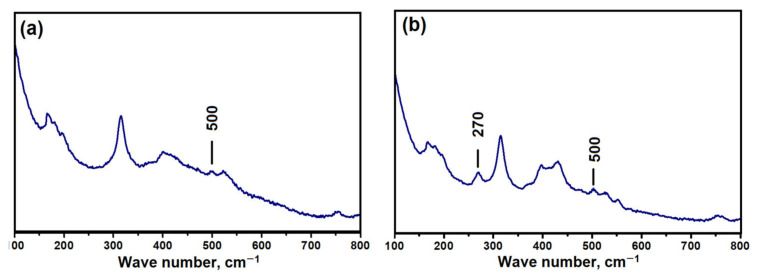
Raman spectra of silicoaluminophosphate gels: (**a**) Sample-SAPO-iAl-1.0; (**b**) Sample-SAPO-iAl-1.5.

As noted above, the gels prepared using aluminum isopropoxide are characterized by the presence of the Al–O–P type bonds. Raman spectroscopy is known to be very sensitive to ring structures forming the molecular sieve lattice. For AlPO_4_-n and SAPO-n molecular sieves, signals at 270 and 500 cm^−1^ are associated with 10R and 4R rings [23,24]. The indicated bands were observed in the spectra of the SAPO-iAl-1.0 and iAl-1.5 samples (Figure 3). For SAPO-iAl-1.5, an additional band was observed at 270 cm^−1^, linked by 10R rings. Thus, future fragments of the SAPO-11 structure are formed at the initial stage of the preparation of the silicoaluminophosphate gel at room temperature.

In summary, the above results revealed that to prepare molecular sieves of high phase purity of the AEL structure, it is necessary that the main phases in the initial gels are aluminophosphates, which have already preformed the Al–O–P type bonds, contributing to the formation of crystals of the SAPO-11 molecular sieve.

The solution to the problem of synthesizing SAPO-11 of high phase purity from initial gels prepared using such sources of Al as hydrated aluminum oxides (boehmite and pseudoboehmite) can be the introduction of an additional aging stage, which will preliminarily form a higher content of aluminophosphates.

**Figure 4:**
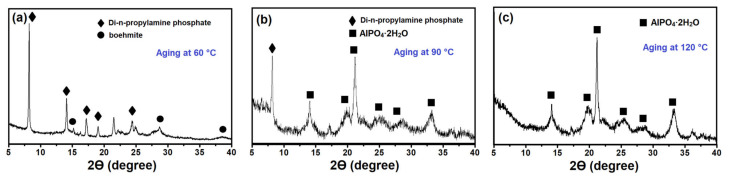
X-ray diffraction patterns of silicoaluminophosphate gels subjected to aging at different temperatures: (**a**) Sample SAPO-PB-1.0(60); (**b**) Sample-SAPO-PB-1.0(90); (**c**) Sample-SAPO-PB-1.0(120).

Figure 4 shows the X-ray diffraction patterns of the gel samples aged at different temperatures. When increasing the aging temperature from 25 to 60 °C, practically no decrease in the intensity of signals characteristic of di-n-propylamine phosphate and undissolved boehmite was observed. The results obtained reveal a weak interaction of the indicated reagents. A further increase in the aging temperature from 90 to 120 °C brings about the appearance of an amorphous phase and hydroaluminophosphate AlPO_4_·2H_2_O in the gels. Introduction of the aging stage at 90 and 120 °C therefore provides formation of predominantly aluminophosphate phases in the initial gels.

**Figure 5:**
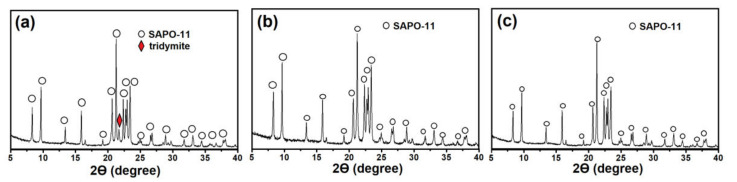
X-ray diffraction patterns of crystallization products of silicoaluminophosphate gels subjected to aging: (**a**) Sample SAPO-11-PB-1.0(60); (**b**) Sample-SAPO-11-PB-1.0(90); (**c**) Sample-SAPO-11-PB-1.0(120).

Figure 5 illustrates X-ray diffraction patterns of gel crystallization products subjected to aging. The gel crystallization products exposed to 90 and 120 °C were found to contain only signals characteristic of SAPO-11. The X-ray diffraction pattern of gel crystallization products aged at 60 °C exhibited additional signals typical for tridymite. Thus, the research results fully confirm our assumption about the need for the presence of aluminophosphate phases in the initial gels with already formed Al–O–P type bonds, providing selective crystallization of SAPO-11.

It is important to note that the results of crystallization significantly depend on the chemical and phase composition of the gels, which determine the pH value. Thus, SAPO-iAl-1.0 SAPO-PB-1.0(120) gel samples and SAPO-PB-1.5 SAPO-PB-1.0(90) gel samples were characterized by pH values of ~7 and ~5, respectively, while the results of their crystallization were significantly different from each other. As will be shown below, both the morphology of the crystals and the characteristics of their porous structure differed significantly.

**Figure 6:**
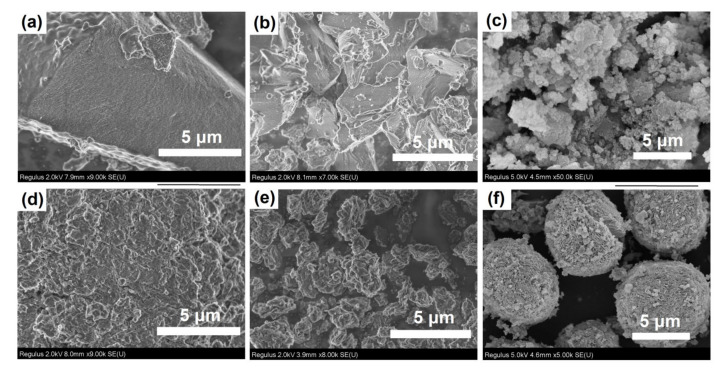
SEM image of silicoaluminophosphate gels: (**a**) Sample SAPO-PB-1.0; (**b**) Sample-SAPO-PB-1.5; (**c**) Sample-SAPO-iAl-1.0; (**d**) Sample-SAPO-iAl-1.5; (**e**) Sample-SAPO-PB-1.0(90); (**f**) Sample-SAPO-PB-1.0(120).

**Figure 7:**
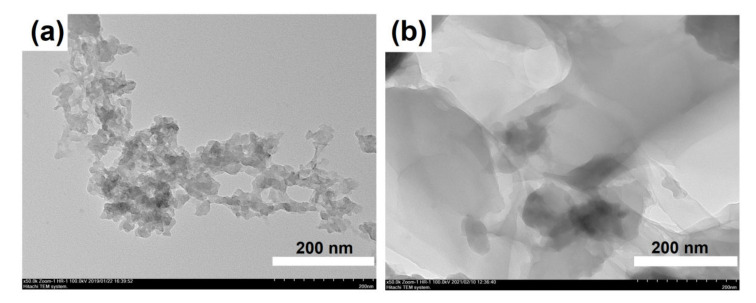
TEM image of silicoaluminophosphate gels: (**a**) Sample-SAPO-iAl-1.0; (**b**) Sample-SAPO-iAl-1.5.

Figure 6 shows the SEM images of dried silicoaluminophosphate gels. SAPO-PB-1.0 is a dense homogeneous material consisting of a mixture of di-n-propylamine phosphate and undissolved boehmite phases, as above-mentioned. The structure of the SAPO-PB-1.5 gel sample with a similar phase composition was composed of aggregates of nanosized particles. The gel samples aged at 90 and 120 °C were spherical aggregates ranging from 10 to 15 μmin in size. Higher magnification revealed that the structure of these materials was similar to a spongy structure, characterized by a developed system of pores ranging from 50 to 200 nmin in size. An amorphous gel sample prepared using aluminum isoproxide SAPO-iAl-1.0 resembled a xerogel in structure. According to the TEM data (Figure 7), the particles were formed of spherical particles of amorphous silicoaluminophosphate ranging from 10 to 20 nm in size. The structure of the SAPO-iAl-1.0 sample seems to be very close to the structure of silica gel and its formation proceeded through the sol–gel synthesis. The aluminophosphate SAPO-iAl-1.5 sample with a layered structure is represented by intergrowths of layers of various thicknesses. The TEM image shows that its structure was formed of thin plates resembling paper glued in several layers.

**Figure 8:**
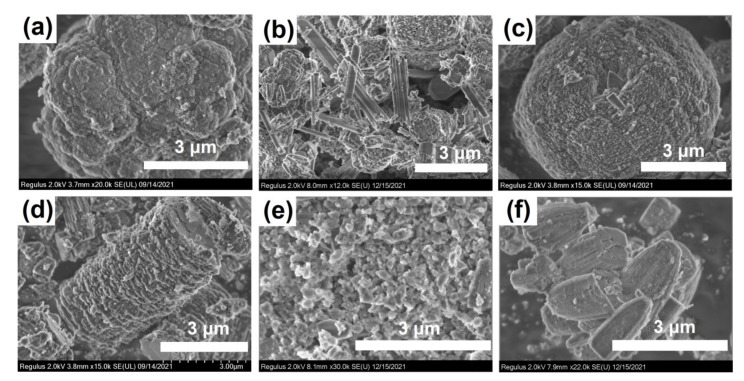
SEM image of crystallization products based on silicoaluminophosphate gels: (**a**) Sample SAPO-11-PB-1.0; (**b**) Sample-SAPO-11-PB-1.5; (**c**) Sample-SAPO-11-iAl-1.0; (**d**) Sample-SAPO-11-iAl-1.5; (**e**) Sample-SAPO-11-PB-1.0(90); (**f**) Sample-SAPO-11-PB-1.0(120).

Figure 8 shows images of gel crystallization products. Crystallization of SAPO-PB-1.0 gel prepared on the basis of boehmite resulted in the formation of SAPO-11 crystals, which are dense spherical aggregates of 6–8 μm in size, consisting of densely packed nanosized crystals. An increase in the content of the template in the gel (SAPO-PB-1.5) brings about the appearance of a mixture of SAPO-11 crystals of various morphologies, namely, elongated needles, spherical intergrowths of nanocrystals, and hexagonal prisms. Furthermore, it should be noted that the introduction of the stage of aging SAPO-PB-1.0 gel at 90 or 120 °C fundamentally changes the morphology of SAPO-11. The SAPO-11-PB-1.0(90) sample was characterized by cubic crystals ranging from 100 to 500 nm in size, while the SAPO-11-PB-1.0(120) sample was characterized by crystals in the form of cones. The SAPO-11 sample prepared from amorphous gel (SAPO-iAl-1.0) was pseudospherical crystal aggregates of 6 to 8 μm in size, consisting of smaller cubic crystals. Moreover, the crystals of the SAPO-11-iAl-1.5 sample obtained from a gel containing a layered phase were aggregates of thin plates forming an elongated cylinder of 6 μm in size. It is noteworthy that the morphology of SAPO-11 crystals in the form of cones and elongated cylinders has not been previously reported.

Thus, the results obtained suggest that by regulating the chemical and phase composition of silicoaluminophosphate gels by using various sources of aluminum in their preparation and changing the content of the template in the reaction mixture as well as by introducing the aging stage, it is possible to control the morphology of primary crystals and secondary aggregates of SAPO-11 molecular sieves.

**Figure 9:**
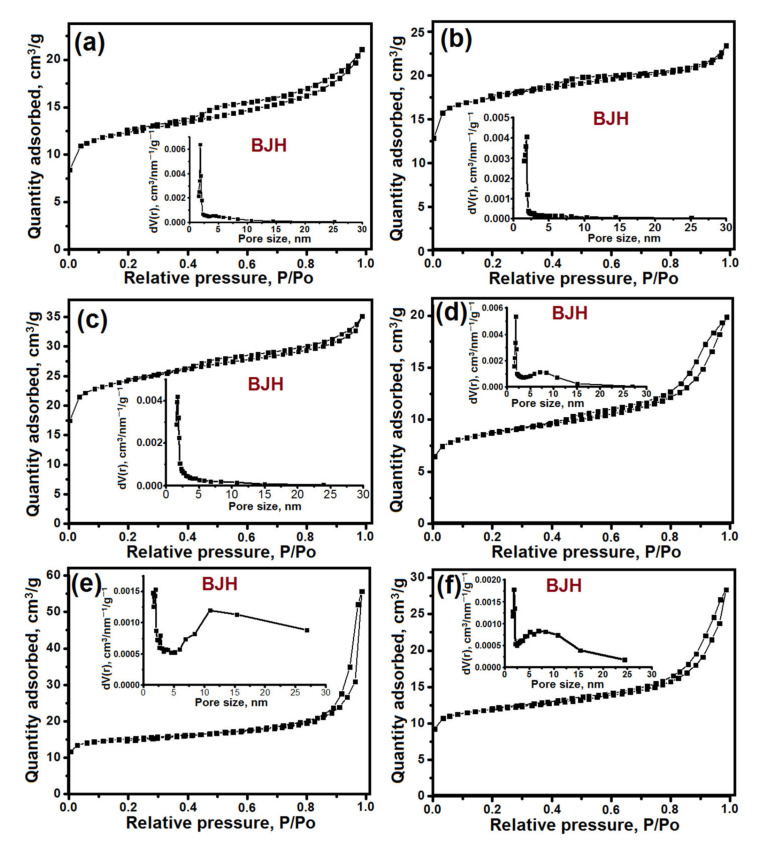
Nitrogen adsorption–desorption isotherms and pore size distribution (BJH) for the crystallization products based on silicoaluminophosphate gels: (**a**) Sample SAPO-11-PB-1.0; (**b**) Sample-SAPO-11-PB-1.5; (**c**) Sample-SAPO-11-iAl-1.0; (**d**) Sample-SAPO-11-iAl-1.5; (**e**) Sample-SAPO-11-PB-1.0(90); (**f**) Sample-SAPO-11-PB-1.0(120).

Figure 9 shows the nitrogen adsorption–desorption isotherms and pore size distribution; Table 3 lists the characteristics of the porous structure of the crystalline silicoaluminophosphate samples. For all SAPO-11 samples, type IV isotherms with a hysteresis loop close to the H4 type were observed. This type of isotherm is typical for micro-mesoporous materials. According to the BJH data, a wide distribution of mesopores of 2 to 25 nm in size was observed for all samples. The SAPO-11-PB-1.0 and SAPO-11-PB-1.5 samples were characterized by the smallest volume of micropores due to the presence of a non-porous tridymite phase in their composition. The introduction of the stage of aging the gel prepared using boehmite at 90 °C allows, upon further crystallization, one to synthesize the SAPO-11 sample with the highest specific surface area and mesopore volume. The results obtained are due to the fact that the secondary porous structure of this sample is formed of crystals ranging from 100 to 500 nm in size, which are only partially fused between.

## 3. Conclusions

The influence of various sources of aluminum (boehmite, Al isopropoxide), the content of the template in the reaction mixture (SDA/Al_2_O_3_ = 1.0–1.5), and as of the temperature (25–120 °C) aging stage on the chemical and phase composition as well as properties of the porous structure of silicoaluminophosphate gels and products of their subsequent crystallization are detailed in the present work by applying the methods of XRD, Raman spectroscopy, SEM, TEM, and N_2_ adsorption–desorption.

It has been established that an regulation of the chemical and phase composition of silicoaluminophosphate gels by changing the reactivity of the aluminum source, the content of the template in the reaction mixture as well as the temperature of the aging stage of silicoaluminophosphate gels makes it possible to address one of the current challenges in the field of the synthesis of molecular sieves with a hierarchical porous structure, namely, to develop approaches for the preparation of SAPO-11 molecular sieves of high phase purity with a hierarchical porous structure without using surfactants and various growth modifiers.

## 4. Materials and Methods

### 4.1. Preparation of Silicoaluminophosphate Gels

For the crystallization of SAPO-11 molecular sieves, we used silicoaluminophosphate gels with the following composition: 1.0Al_2_O_3_·1.0P_2_O_5_·0.3SiO_2_ (1.0;1.5)DPA·40H_2_O. To prepare the gels, Al isopropoxide (iAl, 98%, Acros Organics, Noisy-le-Grand, France) or boehmite (PB, AlO(OH), Sasol SB, Hamburg, Germany), SiO_2_ sol, orthophosphoric acid (H_3_PO_4_, 85%, Reachim, Moscow, Russia), and di-n-propylamine (DPA, 99%, Acros Organics, Schwerte, Germany) were used as sources of Al, P, Si, and a template, respectively. Silicoaluminophosphate gels were prepared as follows: 10.0 g of orthophosphoric acid was added to 27.0 g of distilled water and 17.3 g of Al isopropoxide or 5.6 g of boehmite was added under vigorous stirring to form alumophosphate, then 4.4 or 6.6 g of di-n-propylamine was added to the gel. After DPA was added, the calculated amount of the SiO_2_ source was slowly introduced into the resulting gel, and then the reaction mixture was intensively stirred for 1 h.

Table 4 lists the designations of gel samples prepared using different sources of Al and different contents of the template. The aging temperature of the reaction gels is given in parentheses.

### 4.2. Aging of Silicoaluminophosphate Gels

The samples of SAPO-PB-1.0 silicoaluminophosphate gel were kept in a thermostat at 60, 90, or 120 °C for 24 h. After aging, the gel samples were assigned an additional index (60), (90), and (120), respectively (Table 3).

### 4.3. Crystallization of SAPO-11 Molecular Sieves

SAPO-11 molecular sieves were crystallized from the corresponding silicoaluminophosphate gels at 200 °C for 24 h. Preliminary experiments have shown that crystallization for more than 24 h results in the formation of cristobalite. Table 3 lists the designations of the SAPO-11 samples obtained from silicoaluminophosphate gels of various compositions.

### 4.4. Methods of Material Analysis 

The chemical composition of the obtained silicoaluminophosphate gels and crystallization products was characterized by X-ray fluorescence spectroscopy recorded on a Shimadzu EDX 7000P spectrometer (Duisburg, Germany) by the fundamental parameter method.

Powder X-ray diffraction patterns of silicoaluminophosphate gels and the uncalcined SAPO-11 samples were recorded on a Bruker D8 Advance diffractometer (Karlsruhe, Germany) in CuKα radiation. The patterns were collected over a 2θ range from 5 to 40° with a scanning speed of 1° min. The phase analysis of the obtained X-ray diffraction patterns was performed by the DIFFRAC.EVA program using the PDF2 database. The crystallinity degree was assessed by the content of an amorphous halo in the range from 20 to 30°(2θ). 

Raman spectra of the silicoaluminophosphate gels were obtained on a FT-Raman Thermo Scientific Nicolet NXR 9650 Fourier spectrometer (Waltham, MA, USA) in the range of 70–3800 cm^−1^ with a resolution of 2 cm^−1^. An Nd:YVO_4_ laser with a wavelength of 1064 nm and a power up to 1.5 W was used as an excitation source.

The size and morphological features of silicoaluminophosphate gels and SAPO-11 were detailed by field emission scanning electron microscopy (FE-SEM) with a Hitachi Regulus SU8220 scanning electron microscope (Tokyo, Japan). The images were taken in the mode of registration of secondary electrons at 2 kV accelerating voltage. The microstructure of the samples was also examined by transmission electron microscopy (TEM) on a Hitachi HT 7700 electron microscope (Tokyo, Japan). The images were recorded in the light field mode at an accelerating voltage of 100 kV.

The BET surface area, the volume of micro- and mesopores were measured by the method of low-temperature nitrogen adsorption–desorption with a Nova 1200e sorptometer (Quantachrome Instruments, Boynton Beach, FL, USA). The specific surface area was calculated by the multipoint BET method. The micropore volumes in the presence of mesopores were derived from a *t-*Plot approach. The pore size distribution was calculated by the BJH (Barrett–Joyner–Halendy) model from the desorption branch. Prior to the analysis, fresh samples were calcined at 600 °C for 6 h.

## Figures and Tables

**Table 1:** Chemical and phase composition of silicoaluminophosphate gels.

Sample	Gel	pH Gel	Phase Composition
	Al_2_O_3_·P_2_O_5_·SiO_2_		
SAPO-iAl-1.0	1.00·1.02·0.29	7.1	SAPO
SAPO-iAl-1.5	1.00·0.99·0.28	8.2	Layered.SAPO+SAPO
SAPO-PB-1.0	1.00·1.03·0.29	3.0	Ph.DPA+PB
SAPO-PB-1.0(60)	1.00·1.01·0.30	3.5	Ph.DPA+PB
SAPO-PB-1.0(90)	1.00·1.01·0.29	5.9	Ph.DPA+AlPO_4_·2H_2_O
SAPO-PB-1.0(120)	1.00·1.00·0.29	7.3	AlPO_4_·2H_2_O
SAPO-PB-1.5	1.00·1.02·0.28	5.4	Ph.DPA+PB

Gel—Chemical composition of silicoaluminophosphate gels. SAPO—amorphous silicoaluminophosphate. Layered. SAPO—silicoaluminophosphate with a layered structure. Ph. DPA—di-n-propylamine phosphate. PB—boehmite.

**Table 2:** Chemical and phase composition of crystallization products.

Sample	Cryst.Prod	Phase Composition	DR, %
	Al_2_O_3_·P_2_O_5_·SiO_2_		
SAPO-11-iAl-1.0	1.00·1.01·0.22	SAPO-11	87
SAPO-11-iAl-1.5	1.00·0.94·0.28	SAPO-11+SAPO-41	90
SAPO-11-PB-1.0	1.00·0.99·0.14	SAPO-11+Tr	-
SAPO-11-PB-1.0(60)	1.00·0.98·0.16	SAPO-11+Tr	-
SAPO-11-PB-1.0(90)	1.00·0.96·0.22	SAPO-11	95
SAPO-11-PB-1.0(120)	1.00·0.97·0.19	SAPO-11	97
SAPO-11-PB-1.5	1.00·0.98·0.17	SAPO-11+Tr	-

Cryst.Prod—crystallization products. Ph. DPA—di-n-propylamine phosphate. PB–boehmite. Tr—tridymite. DR—degree of crystallinity.

**Table 3:** Characteristics of the porous structure of crystalline silicoaluminophosphates.

Sample	S_BET_, m^2^/g	S_EX_, m^2^/g	V_micro_, cm^3^/g	V_meso_, cm^3^/g
SAPO-11-PB-1.0	196	110	0.04	0.08
SAPO-11-PB-1.0	245	112	0.06	0.04
SAPO-11-PB-1.0(90)	250	120	0.07	0.27
SAPO-11-PB-1.0(120)	190	81	0.05	0.13
SAPO-11-iAl-1.0	250	127	0.07	0.06
SAPO-11-iAl-1.5	203	109	0.05	0.13

S_BET_—BET surface area. S_EX_—external area. V_micro_—micropore volume. V_meso_—mesopore volume.

**Table 4:** Conventional designation of silicoaluminophosphate gels of various chemical composition and crystallization products.

Sample	Source Al	Gel Composition
SAPO-iAl-1.0	iAl	1.0Al_2_O_3_·1.0P_2_O_5_·0.3SiO_2_·1.0DPA·40H_2_O
SAPO-iAl-1.5	iAl	1.0Al_2_O_3_·1.0P_2_O_5_·0.3SiO_2_·1.5DPA·40H_2_O
SAPO-PB-1.0	PB	1.0Al_2_O_3_·1.0P_2_O_5_·0.3SiO_2_·1.0DPA·40H_2_O
SAPO-PB-1.0(90)	PB	1.0Al_2_O_3_·1.0P_2_O_5_·0.3SiO_2_·1.0DPA·40H_2_O
SAPO-PB-1.0(120)	PB	1.0Al_2_O_3_·1.0P_2_O_5_·0.3SiO_2_·1.0DPA·40H_2_O
SAPO-PB-1.5	PB	1.0Al_2_O_3_·1.0P_2_O_5_·0.3SiO_2_·1.5DPA·40H_2_O
SAPO-11-iAl-1.0	iAl	1.0Al_2_O_3_·1.0P_2_O_5_·0.3SiO_2_·1.0DPA·40H_2_O
SAPO-11-iAl-1.5	iAl	1.0Al_2_O_3_·1.0P_2_O_5_·0.3SiO_2_·1.5DPA·40H_2_O
SAPO-11-PB-1.0	PB	1.0Al_2_O_3_·1.0P_2_O_5_·0.3SiO_2_·1.0DPA·40H_2_O
SAPO-11-PB-1.5	PB	1.0Al_2_O_3_·1.0P_2_O_5_·0.3SiO_2_·1.5DPA·40H_2_O

## Data Availability

Not applicable.
